# Bevacizumab-loaded CalliSpheres beads: *in vitro* loading, release profiles and application in rabbit liver VX_2_ tumor model

**DOI:** 10.3389/fonc.2023.1153759

**Published:** 2023-07-19

**Authors:** Kewei Ren, Yahua Li, Zihe Zhou, Kunpeng Wu, Jianan Wang, Jianning Yao, Yifan Li, Xiaoyong Ge, Xiao Li, Zhen Li, Zongming Li, Xinwei Han

**Affiliations:** ^1^ Department of Interventional Radiology, The First Affiliated Hospital of Zhengzhou University, Zhengzhou, China; ^2^ Engineering Technology Research Center for Minimally Invasive Interventional Tumors of Henan Province, Zhengzhou, Henan, China; ^3^ Department of Gastroenterology, The First Affiliated Hospital of Zhengzhou University, Zhengzhou, China; ^4^ Interventional Institute of Zhengzhou University, Zhengzhou, China

**Keywords:** bevacizumab, Callispheres beads, TACE, VEGF-A, liver VX_2_ tumor

## Abstract

**Background:**

Bevacizumab loaded drug-eluting beads have the potential to reduce TACE related VEGF expression. The purpose of this study was to investigate the in vitro loading, and release profiles of bevacizumab (BEV) loaded on Callispheres beads (CB) and its application in rabbit liver VX_2_ tumor model.

**Methods:**

CB with sizes of 100-300 um and 300-500 um were divided into 5 groups, respectively. BEV with different content was prepared for CB loading, releasing and detected in the solution at different time points. The diameters of CB in each group were measured under a light microscope to calculate the shrinkage rate. The rabbit with VX2 liver model were divided into control group, CB-TACE group, CB-TACE+BEV group, and BEV group. The data of blood test, CT image, HE and IHC staining were compared and analyzed.

**Results:**

The shrinkage rate of the 100-300 um CB was 2.6-7.2%, while the 300-500 um CB was 0.2-7.1%. The BEV-loaded CB (BEV-CB) has a burst release during the first hour and following gradually released with time. The release profiles of 100-300 um CB reach 34% in 24 hours, while the 300-500 um CB to 25.8%. BEV-CB with sizes of 100-300 um was chosen to perform transcatheter arterial chemoembolization (TACE). The results showed that BEV-CB-TACE not only gradually increased the content of BEV in serum and organ tissue but also reduced the level of VEGF in serum. Pathological results suggested that the expression of HIF-1 was elevated while VEGF and MVD decreased when compared to the other groups.

**Conclusion:**

In conclusion, this study confirms that Callispheres beads could efficiency loaded BEV. BEV-CB-TACE has a good safety and effectiveness, and its application could reduce the level of VEGF-A in serum in the treatment of VX_2_ tumors.

## Background

Hepatocellular carcinoma (HCC) is the sixth most common tumor, and the tumor mortality rate ranks third worldwide (1). However, almost 2 to 3 of HCC patients lost the opportunity to receive surgical resection when diagnosed. Thus, transarterial chemoembolization (TACE) has become the standard treatment for advanced HCC (2). TACE has both the advantages of chemotherapy and embolization, but it also causes inner-tumor hypoxic changes, which leads to the up-regulation of vascular endothelial growth factor (VEGF) levels. In turn, the up-regulated VEGF promotes the formation of new tumor-feeding arteries and affects the therapeutic effect (3). Previous studies have demonstrated that the up-regulated VEGF level significantly correlated with the poor prognosis of patients after TACE (4, 5). Although applying drug-eluting beads (DEB) during the TACE procedure increases the local concentration of drugs in tumor tissues, reducing VEGF secretion and tumor angiogenesis is still the key to solving the problem of tumor recurrence (3).

Bevacizumab (BEV) is a monoclonal antibody that blocks VEGF-A. In a phase II study, a two-week regimen of 5-10 mg/kg BEV in HCC patients showed significant antitumor effects, with an ORR of 13%, and 65% of patients were progression-free at 6 months, but serious bleeding occurred in 11% of patients (6). How to reduce the side effects of BEV in the process of tumor treatment is essential in the treatment of HCC.

The study of CHANCE001 demonstrated that TACE plus PD-(L)1 blockades and Tyrosine Kinase Inhibitor (TKI) could significantly improve PFS, OS, and ORR versus TACE monotherapy for Chinese patients (7, 8). The application of DEB with TKI during TACE can effectively inhibit the level of VEGF in serum. Previous studies have successfully applied the combination of DEB and TKI to rabbit VX2 tumor models, which shows good outcomes (9–11). Callispheres beads (CB) is a kind of domestic DEB. However, no studies explored the results of CB loaded BEV (BEV-CB) application in liver tumors. Therefore, this study aims to investigate the *in vitro* loading, release profiles of BEV loaded on CB, and the feasibility and effectiveness of BEV-CB in the TACE procedure.

## Materials and methods

All materials were purchased and used without further purification. Callispheres beads were purchased from Hengrui company, Jiangsu province, China. Bevacizumab ELISA kit was purchased from Shanghai Ruifan Biotechnology Co., Ltd. BCA kit was Shanghai purchased from Epizyme Biomedical Technology Co., Ltd. Bevacizumab was purchased from innoventbio Jiangsu province, China.

### BEV loading and release profiles

CB in the size of 100-300 um and 300-500 um were divided into 5 groups, respectively. Each group contained 0.1 g CB. The 4 ml BEV concentration with 2 mg, 4 mg, 6 mg, 8 mg, and 10 mg contained was given to each group. After CB loading 10 min, 20 min, 30 min, 40 min, 50 min, and 60 min, 20 ul of the supernatant solution was aspirated for BEV concentration detection, and then supplemented with an equal volume of deionized water. After 60 min of loading, the CB in each group were screened through a 70 um cell sieve, rinsed with 1 ml of sterile water, and then placed in 20 ml of normal saline for BEV release profiles. After 10 min, 20 min, 30 min, 40 min, 50 min, 60 min, 2 h, 3 h, 6 h, 12 h, and 24 h release, 20 ul of the supernatant solution was aspirated for BEV concentration detection, and then supplemented with an equal volume of normal saline. The BCA method was used to detect BEV content.

### Morphological properties

CB in the size of 100-300 um and 300-500 um were divided into 5 groups, respectively. Each group contained 0.1 g CB. The 4 ml BEV concentration with 2 mg, 4 mg, 6 mg, 8 mg, and 10 mg content was given to each group. After the solution was added for 40 min, the CB was divided into two parts, which were used for observation and evaluation by ordinary light microscope and scanning electron microscope (SEM) respectively. The diameters of unloaded and loaded beads were measured respectively, 200 beads were counted in each group, and the shrinkage rate was calculated. The shrinkage percentage was calculated as follows: Percentage of shrinkage rate (%) = (average diameter before drug loading - average diameter after drug loading)/average diameter before drug loading*100%.

The CBs with size of 300-500 um loaded BEV were added into the embedded agent to perform frozen section. The slide with slices of CBs were used to perform SEM and energy dispersive spectroscopy (EDS).

### Animal study

This study was carried out with the Guidelines on the Care and Use of Laboratory Animals issued by the Chinese Council on Animal Research and the Guidelines of Animal Care. The protocol was approved by the Committee on the Ethics of Animal Experiments of our institution. Animal studies were conducted at Henan Key Laboratory for Pharmacology of Liver Disease. All rabbits were maintained in single cages at a room temperature of 22 ± 2°C, a relative humidity of 45 ± 15% and a 12-h light/dark cycle. Standard food and water could be accessed freely.

### The establishment and imaging evaluation of rabbit liver VX_2_ tumor model

Thirty New Zealand white rabbits weighted 3.0 ± 0.2 kg were selected, and both males and females were used for the establishment of the liver VX**_2_** tumor model. After successful anesthesia, the dorsal skin of the abdomen was sterilized and a drape was applied, and the abdominal tissue was incised layer by layer along the lower part of the sternum to expose the left lobe of the liver. The tumor tissue of the tumor-bearing rabbit was obtained and chopped into pieces of 1 mm^3^. A sharp knife was used to pierce the wound with a depth of about 1 cm in the left lobe of the liver, the tumor tissue was buried, and a gelatin sponge was used to incarcerate to stop the bleeding. Then, the abdomen was closed layer by layer, and the animals were reared in the animal room for 2 weeks. Then, enhanced CT and contrast-enhanced ultrasound were performed to evaluate the tumor.

### Treatment procedure

The rabbits with liver VX2 tumors were randomly divided into 5 groups (n = 6). The control group received shame TACE, saline was injected instead of the embolization agent during the procedure. The CB-TACE group received CB without drug-loaded embolization during the TACE procedure. The CB-TACE+BEV group received CB-TACE and BEV intravenous injections. The BEV-CB-TACE group received CB with BEV-loaded embolization during the TACE procedure. The BEV group received shame TACE and BEV intravenous injection.

The BEV-loaded CB was prepared using an aseptic technique. One gram of CB was administered with BEV 100 mg/4 ml, and it was kept for 40 min before use. The dosage of BEV intravenous injection was defined as 5 mg/kg according to instructions and finished injection at least 20 min.

After the rabbit with VX**_2_** liver tumor was successfully anesthetized, the skin was prepared, disinfected, draped, and incised to expose the femoral artery. A 22G puncture needle was used to puncture the femoral artery and placed a 4F arterial sheath. A 2.7F coaxial microcatheter was introduced through the sheath to super-select the hepatic artery. Angiography was performed to identify the arteries supplying the VX**_2_** tumor. Before embolization, the BEV-CB solution was mixed with an equal proportion of contrast agents. The time to stop embolization was determined by the blood flow stasis. Repeat angiography to confirm the complete embolization. Withdrawn the microcatheter, pulled out the catheter sheath, and the femoral artery was ligated and the skin was sutured. After the rabbit recovered from anesthetizing, they were sent to the animal cage.

### Blood sample acquisition and testing

One ml of blood before TACE and after TACE 1 d, 3 d, 7 d, and 14 d was obtained from each rabbit. After centrifugation, the supernatant was taken and stored at -80°C for further use. The levels of ALT, AST, BUN, BEV, and VEGF-A in serum were detected by ELISA assay.

### Computed tomography evaluation

Enhanced computed tomography (CT) was used to evaluate the results of treatment. The longest diameter (D) and shortest diameter (d) of each tumor were measured on the arterial phase image. The tumor volume (V) was defined as V= 0.5*D*d^2^. The tumor growth rate was defined as the ratio of pre-treatment volume and post-treatment volume.

### Pathological specimen acquisition

The rabbits were sacrificed on 7 d and 14 d after treatment, respectively. the heart pex, left lobe liver with tumor, spleen, left lung apex, and left lower pole of left kidney were obtained and stored in a -80°C refrigerator for the detection of BEV content. Liver VX**_2_** tumor specimens were obtained and fixed with paraformaldehyde for HE staining, TUNEL immunofluorescence staining, and immunohistochemical staining of HIF-1, VEGF, PCNA, and CD31.

### Statistical analysis

The measurement data were described as mean ± standard deviation. The diameter of CB before and after drug loading was compared by t-test. The BEV concentration in different organs and tissues was compared by one-way ANOVA, and multiple comparisons were determined by the LSD test. Figures were made by GraphPad Prism 7.00 software. Statistical analysis was performed using SPSS 21.0, and P < 0.05 was considered statistically significant.

## Results

### Morphological properties of CB before and after BEV loading

**Figure 1:**
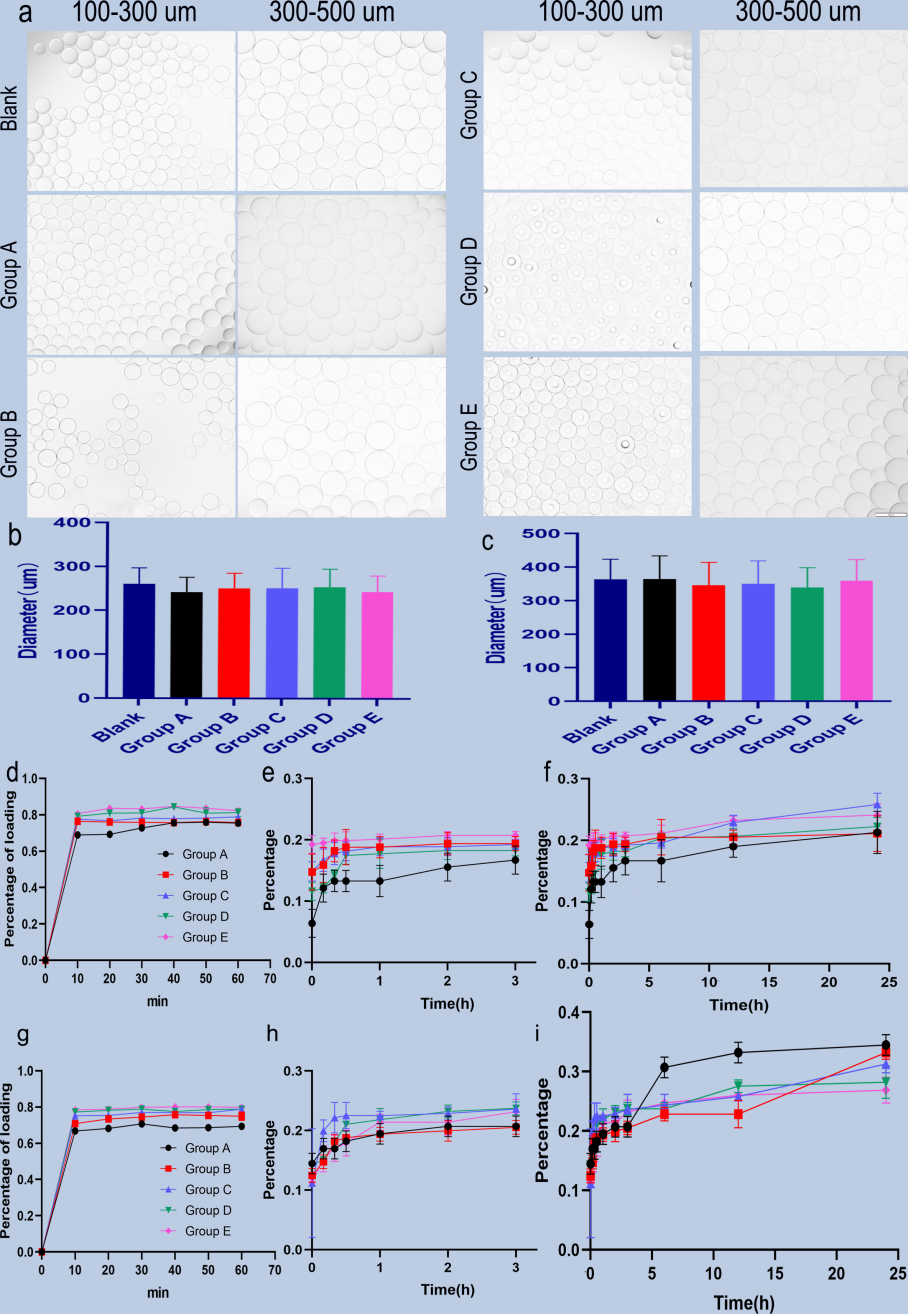
Microscopic view of BEV-loaded CB and BEV loading and releasing. **(A)** 100-300 um and 300-500 um CB were loaded with BEV under a light microscope with 100x magnification image, scale bar is 100um. **(B, C)** The diameter comparison of 100-300 um and 300-500 um beads with BEV respectively. **(D)** 100-300 um CB BEV adsorption diagram within 60 min, **(E, F)** 100-300 um CB after adsorption of BEV release diagram within 3 h and 24 h. **(G)** 300-500 um CB BEV adsorption diagram within 60 min. **(H, I)** 300-500 um beads after adsorption of BEV release diagram within 3 h and 24 h.

The images of two-size CB before and after different content BEV loading are shown in **Figure 1A**. The measurements of diameter are shown in **Figures 1B, C**. The two-size CB showed different degrees of shrinkage after loading with different BEV concentrations. The shrinkage rate in CB with 100-300 um ranges from 2.6% to 7.2%, while the CB with 300-500 um from 0.2% to 7.1% (**Table 1**).

**Table 1:** The shrinkage ration of two sizes CB.

	Diameters (µm)					
Size ranges	Blank	Group A	Group B	Group C	Group D	Group E
100µm	260.84 ± 36.00	242.16 ± 33.70	250.87 ± 34.14	251.52 ± 45.16	254.00 ± 40.94	243.29 ± 36.53
Diameter Shrinkage ratio		7.2%	3.8%	3.6%	2.6%	6.7%
300µm	362.95 ± 60.36	362.37 ± 69.37	340.59 ± 67.37	349.95 ± 68.92	337.03 ± 61.18	359.10 ± 62.67
Diameter Shrinkage ratio		0.2%	6.1%	3.6%	7.1%	1.1%

**Figure 2:**
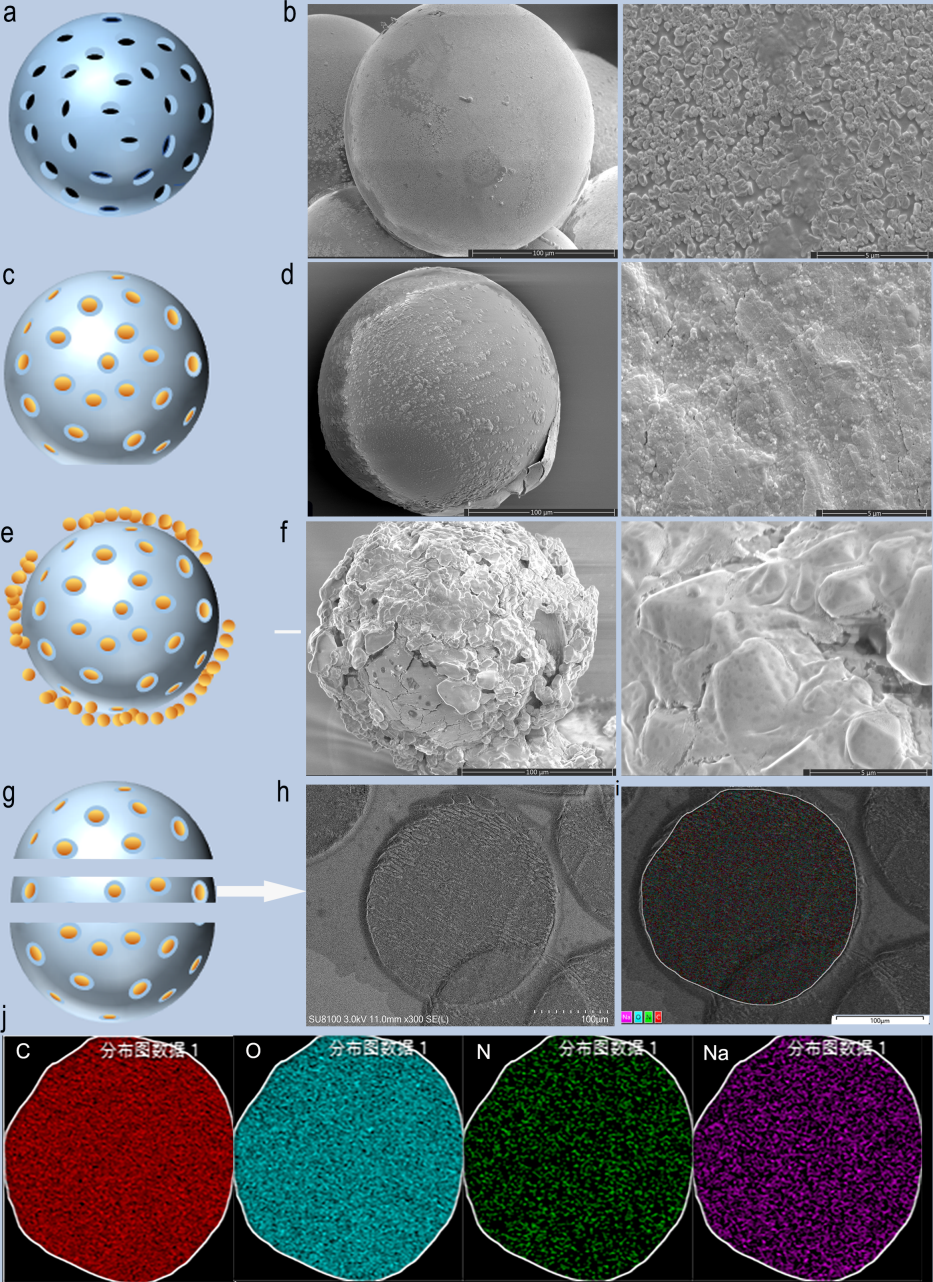
SEM images and schematic diagram of CB loaded with BEV. **(A, B)** Schematic diagram and SEM images of blank CB. **(C, D)** Schematic diagram and SEM images of BEV-loaded CB. **(E, F)** Schematic diagram and SEM of BEV-loaded CB after 1-week drying. **(G, H)** BEV-CB frozen section diagram and SEM image. **(I, J)** EDS analysis of BEV-CB. C, carbon; O, oxygen; N, nitrogen; Na, sodium.

The scanning electron microscope (SEM) results of CB show that the surface structure is relatively smooth before loading. Irregular pore-like structures on the surface of CB were observed with magnification (**Figures 2A, B**). After BEV loading, the outer surface of CB was covered with a layered structure and partially peels off. The magnification SEM images of CB showed that the layered structure on the surface of the bead is dense. The irregular pore structures shown on unloaded CB were not visible, which were replaced by denser BEV coating (**Figures 2C, D**). After drying, obvious shrinkage and fragmentation of CB were observed on the SEM image. The fragmented crystal structure can be observed after magnification (**Figures 2E, F**).

A single 300-500 um bead was successfully sliced with a thickness of 30 um, and then SEM and EDS analysis were performed (**Figures 2G, H**). The results showed that the main element was C, O, N, and Na (**Figures 2I, J**). The component of CB was polyvinyl acetate and the main element was C and O. The CB was stored in 0.9% NaCl solution. while the N element comes from BEV. Thus, the result strongly suggested that the BEV was loaded in the inner structure of CB. According to the results of SEM, it can be speculated that the process of BEV loading of CB is ion exchange combined with surface adsorption. The schematic diagram was shown in **Figures 2A, C, E,** and **G**.

### BEV loading and releasing by CB

BEV loading: The adsorption of two-size CB in different BEV concentrations is shown in **Figures 1D, G**. The maximum adsorption rate of the 100-300 um CB was (84.66 ± 0.61) % and the time to reach the maximum adsorption rate was 40 minutes after loading. while the maximum adsorption rate of 300-500 um CB was (80.16 ± 0.32)% and the time to reach the maximum adsorption rate was also 40 min. In addition, for the loading of CB in different BEV concentration solutions, the minimum time for 100-300 um CB to reach the maximum loading rate is 40 min, and the minimum time for 300-500 um CB to reach the maximum loading rate is 30 min. However, after the CB was loaded for 10 min, the loading efficiency was similar to the maximum loading efficiency. It is 91.2%-99.7% in the 100-300 um group and 93.5%-98% in the 300-500 um group (**Table 2**).

**Table 2:** The loading efficiency of two sizes CB.

Diameter range (um)	BEV content (mg)	Loading efficiency at 10 min	Maximum loading efficiency (%)	Time to maximum loading (min)
**100-300**	20	69.29 ± 0.78	75.95 ± 1.64	50
**300-500**		66.78 ± 1.39	70.71 ± 1.6	30
**100-300**	40	76.07 ± 0.52	76.27 ± 0.28	50
**300-500**		70.86 ± 1.02	75.77 ± 1.34	40
**100-300**	60	77.6 ± 0.22	78.85 ± 0.26	60
**300-500**		75.2 ± 0.28	78.91 ± 1.62	60
**100-300**	80	79.22 ± 1.35	84.66 ± 0.61	40
**300-500**		78.84 ± 0.87	80.16 ± 0.32	40
**100-300**	100	80.6 ± 1.05	84.33 ± 1.18	40
**300-500**		77.31 ± 1.59	78.88 ± 0.78	30

BEV releasing: The CB with BEV have a burst release in the first hour. Followed gradually increasing. The BEV releasing in CB with 100-300 um can reach 34% in 24 hours, while 300-500 um CB can reach 25.8% (**Figures 1E, F, H, I**).

### Animal study

**Figure 3:**
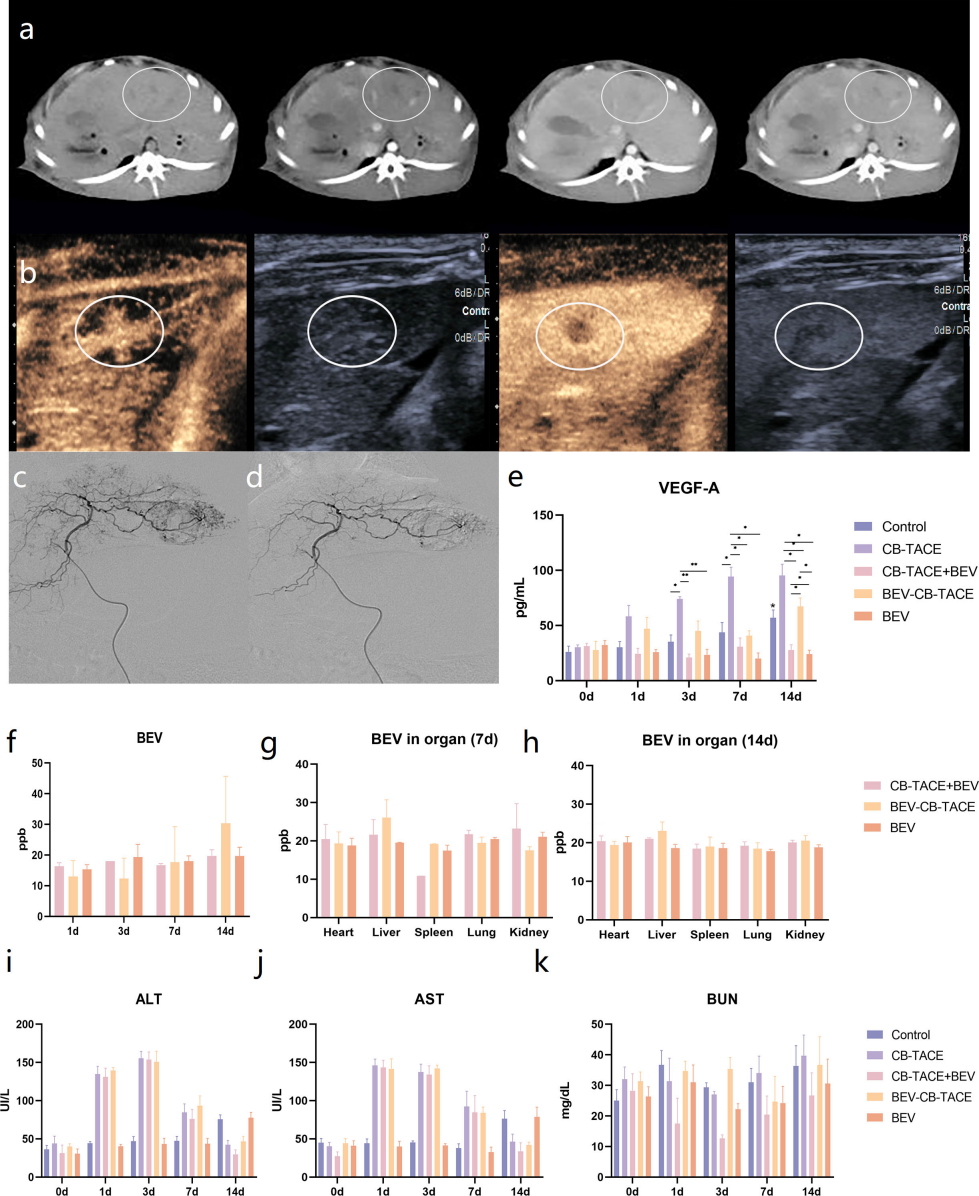
Rabbit liver VX2 tumor model evaluation and treatment. **(A)** CT scan and three-phase enhancement could observe low-density lesion in the left part of the liver. **(B)** Contrast-enhanced ultrasound suggested the obvious phenomenon of “fast in and fast out”. **(C)** Hepatic arteriography revealed that the hypervascular lesion originate from the left hepatic artery. **(D)** The abnormal vascular disappeared after TACE. **(E)** Changes of VEGF-A content in serum pre-treatment and post-treatment 1 d, 3 d, 7 d, and 14 d. **(F)** Changes of BEV content in serum post-treatment 1 d, 3 d, 7 d, and 14 d. **(G)** The content of BEV in the heart, embolized liver, spleen, lung, and kidney post-treatment 7 d and 14 d. **(H-K)** Changes of ALT, AST, and BUN content in serum pre-treatment and post-treatment 1 d, 3 d, 7 d, and 14 d. (*P < 0.05; **P < 0.01).

The rabbit VX**_2_** liver tumor model was successfully established. Intrahepatic low-density shadows were observed on a plain CT scan. Slightly high-density enhancement around the tumor was observed in the enhanced arterial phase. Intrahepatic low-density mass was also observed in the venous and delayed phases (**Figure 3A**). The typical fast-in and fast-out behavior were observed on CEUS (**Figure 3B**).

The hepatic artery angiography before and after tumor embolization are shown in (**Figures 3C, D**). The tumor-feeding artery originated from the left hepatic artery and encircled the tumor in a ring, representing a significant “ball-holding” sign. After embolization, blood flow has been abolished. During the operation, the dosage of 100-300 um BEV- CB was 0.4 ml. No procedure-related complications happened.

Blood testing results indicated that the level of VEGF-A after treatment gradually increased in the group of control, CB-TACE, and BEV-CB-TACE, while slightly decreasing in CB-TACE and BEV groups (**Figure 3E**). The content of BEV in serum was detected immediately after treatment and post-treatment 1 d, 3 d, 7 d, and 14 d. The results showed that serum BEV levels gradually increased in the BEV-CB-TACE group, while no obvious change in CB-TACE+BEV and BEV groups (**Figure 3F**). Post-treatment 7 days, the BEV content in the embolized liver in the BEV-CB-TACE group was significantly higher than that in the heart, spleen, lung, and kidney. While the distribution of BEV in the heart, spleen, lung, and kidney with no statistical difference in CB-TACE+BEV and BEV groups (**Figure 3G**). At 14 days, the distribution of BEV in the heart, spleen, lung, and kidney with no statistical difference in the CB-TACE+BEV, BEV-CB-TACE, and BEV groups (**Figure 3H**).

The level of ALT and AST were increased in the group of CB-TACE, CB-TACE+BEV, and BEV-CB-TACE from post-treatment 1 day to 7 days and reduced to normal range on post-treatment 14 days. The level of ALT and AST gradually increased in the control group, while, fluctuating within the normal range in the BEV group (**Figures 3I, J**). The level of BUN fluctuated within the normal range in all 5 groups (**Figure 3K**).

**Figure 4:**
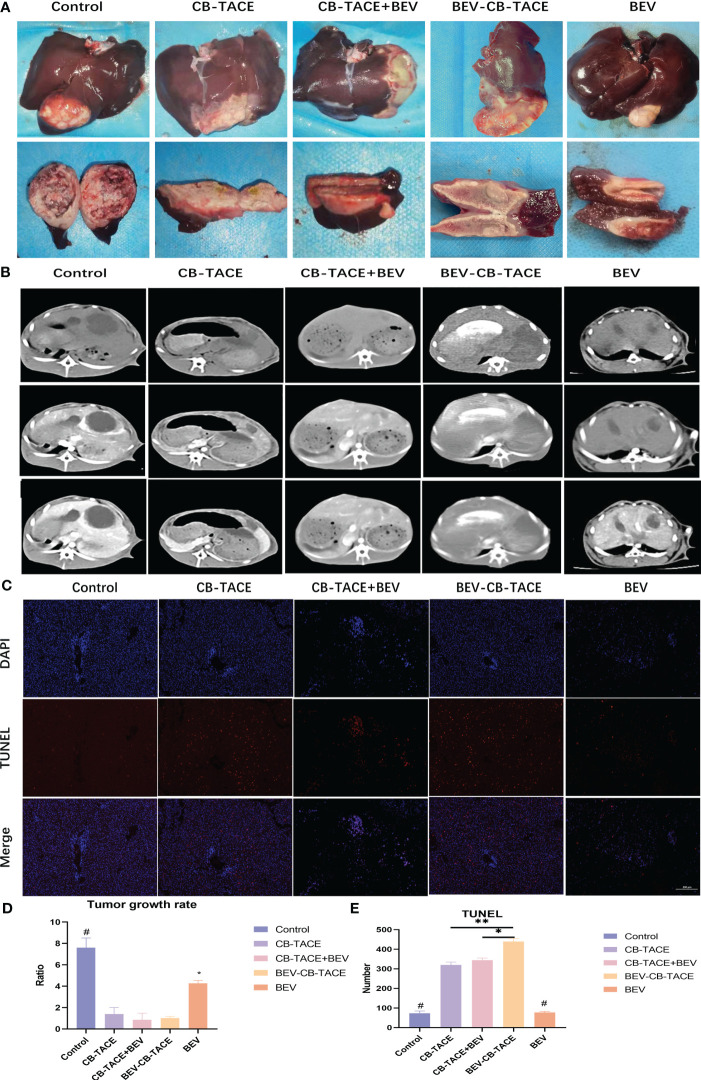
The results of rabbit liver VX2 model post-treatment 14d. **(A)** Macroscopic view of rabbit liver VX2 tumor post-treatment 14 d. **(B, D)** Represent enhanced CT evaluate the tumor growth post-treatment 14 d. **(C, E)** TUNEL immunofluorescent staining evaluates the tumor cell necrosis post-treatment 14 d. (*P < 0.05; **P < 0.01). P<0.05 compared to BEV-CB-TACE.

After embolization, the macroscopic view of the liver showed gray-yellow necrosis in the liver lobes and tumor tissue (**Figure 4A**). The represent 3 phase-enhanced computed tomography images of 5 groups were shown in **Figure 4B**. The tumor growth rate in the control group was significantly higher than the other 4 groups, while in the BEV group was higher than the other 3 groups except for the control group. The tumor growth rate with no statistical differences among CB-TACE, CB-TACE+BEV, and BEV-CB-TACE groups (**Figure 4D**). The TUNEL immunofluorescent staining images were shown in **Figure 4C**. The number of apoptosis cells in the BEV-CB-TACE group was significantly higher than in the other 4 groups. While there was no statistical difference between CB-TACE and CB-TACE+BEV groups (**Figure 4E**).

**Figure 5:**
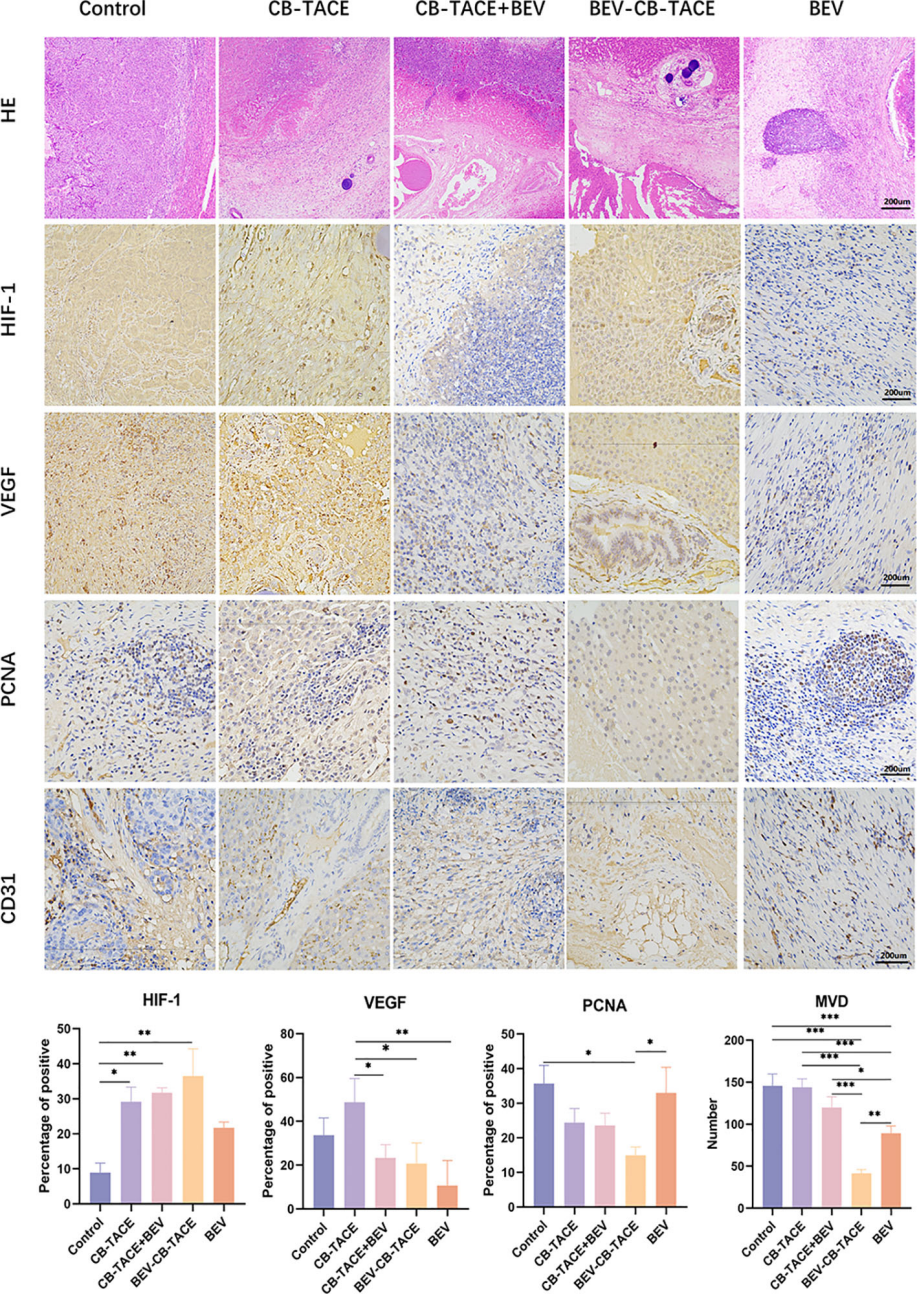
Representative HE and IHC demonstrating HIF-1, VEGF, PCNA, and CD31 (count for MVD) staining in rabbit liver VX2 model post-treatment 14 d. (*P < 0.05; **P < 0.01; ***P < 0.001).

After embolization 2 weeks. The HE staining showed that the tumor arteries were embolized by CB, as well as necrotic tumor tissue and part of normal liver tissue (**Figure 5**). The IHC staining of HIF-1 result showed that the expression of HIF-1 in the control group was lower than in the other 4 treatment groups, especially in the 3 groups with arterial embolization. The percentage of HIF-1 positive in the BEV-CB-TACE group was higher than CB-TACE and CB-TACE+BEV groups. The expression of VEGF in the CB-TACE group was higher than in the other 4 groups. The percentage of VEGF positive in BEV using group is lower than the control group and CB-TACE group. The control group and BEV group with a higher percentage of PCNA positive expression. As a maker of MVD, CD 31IHC staining was used to count the number of MVD. The number of MVD in the BEV-CB-TACE group was significantly lower than in the other 4 groups. The control group with the highest number of MVD.

## Discussion

TACE is the first-line treatment for unresectable liver cancer. The application of drug-eluting beads in TACE shows a better effect than lipiodol, which can continuously release a higher concentration of chemotherapeutic drugs locally in the tumor when embolizing the tumor (12). The recurrence of liver cancer after TACE was associated with neovascularization of the embolized tumor (2). BEV is a monoclonal antibody that blocks VEGF-A. The combination of drug-eluting beads and BEV for HCC local treatment hasn’t been explored. Therefore, we designed this study to explore the loading and releasing profiles of BEV on CB. as well as the results of BEV-loaded CB TACE in rabbits with liver VX2 tumors.

In this study, we found that CB showed a good result in BEV loading. The maximum loading rate of 100-300um CB could reach (84.66 ± 0.61) %, and the maximum loading rate of 300-500um CB could reach (80.16 ± 0.32) %. The loading efficiency of CB is related to CB size and BEV concentration. The release of BEV-loaded CB in saline solution showed a process of burst release within the first 1 hour, and only 30% BEV of the release was completed within 24 hours. The results of the animal study showed that the application of CB-loaded BEV in the procedure of TACE could reduce the level of serum VEGF.

CB showed different degrees of shrinkage after loading BEV. Previous studies have shown that different degrees of shrinkage will occur after CB is loaded with drugs, and the shrinkage degrees of different drugs are also different (13). After CB was loaded with 80mg irinotecan, the shrinkage rates of 50-150 um, 100-300 um, and 300-500 um were 8.5%, 10.9%, and 16.2%, respectively. At present, no research reported CB loaded with BEV. For other commercially available beads, only QSM and DC beads have been reported (6). The results of a dose of QSM loaded with 200 mg BEV showed that the maximum loading rate was 59% after 90 minutes. Released 52% in one hour and 68% in 16 hours. Some studies have shown that the loading efficiency of CB can reach 33% in the first 5 minutes of loading, and only 46% in the following 2 hours (14). Our results showed that the loading rate of CB on BEV was higher than that of QSM and DC, and the drug release time was also longer than that of QSM. DC beads did not explore the drug release profile after BEV loading. The loading of BEV by DC beads is mainly carried out by electrostatic adsorption (14–17). The molecular of BEV brings a positive charge, while the sulfonate groups in the beads carry a negative charge after ionization (18, 19). Therefore, electrostatic attraction is the driving force for the adsorption of BEV molecules on the surface of the beads. While CB is mainly loaded by ion exchange, there is also surface adsorption. From the results of BEV loading and releasing profiles, we speculate that there is a dynamic equilibrium process in the adsorption and release process of the beads. The EDS of the beads also confirms the coexistence of Na and N elements, that is, there are a large number of Na ions and BEV molecules in the beads at the same time, and also indicated that the release of BEV was also related to ion exchange. The process that affects the release of BEV may be related to the content of Na ions, or positively charged substances. Since the solvent used in this release experiment is 0.9% NaCl, it may lack protein and other related biological macromolecules in the human body, so it may have a certain impact on the release of BEV.

In this study, the shrinkage of CBs with different diameters and different contents of BEV was different. 300-500 um CB showed no statistically significant difference in shrinkage rate among different contents of BEV, while 100-300 um CB showed a significant statistical difference in group A and group E, but there was no statistical difference in group B, C, and D, when compared with group A. These changes may be related to the diameter of the beads. The main reason is that the sizes of the beads are different and exist in a certain range, and the selection in the counting process determines the existence of errors. Studies have shown that the shrinkage rate of CB is smaller than that of Hepasphere and DC beads after loading with drugs (13, 20–22). This change may be caused by the interaction between the hydrogel matrix and the hydrophobic drug inside the beads to replace the water content of the beads.

Different from small-molecule chemotherapeutic drugs, after BEV-loaded CB application, with the release of BEV, the content of BEV in serum also gradually increased. Due to the long half-life of BEV, the content of BEV in serum was not observed a downward trend 14 days after embolization. The BEV content in serum showed a gradually increasing performance after embolization from 1 day to 14 days. While, in CB-TACE+BEV and BEV groups, the level of serum BEV is almost the same as post-application 1 day. The previous study about CB-loaded small-molecule chemotherapeutic drugs suggested that after D-TACE, the content of doxorubicin in serum reached a peak in 1-3 days, and then gradually decreased, and a high serum drug concentration could only be maintained for 7 days (11, 20, 22). The study found that serum BEV maintained a higher concentration for more than 14 days after CB-loaded BEV embolization. Moreover, the distribution of BEV in the embolized liver tissue was higher than in the other organs post-surgery 7 days. After embolization for 14 days, the distribution of BEV in organs is almost the same level. Which is related to the release of BEV from CB and BEV redistribution. It is the main difference between the BEV-CB-TACE group and BEV intravenous application group (CB-TACE group and BEV group).

The reoccurrence of HCC after embolization is a challenging disease. Hypoxia-inducible factor (HIF)-mediated various molecular mechanisms contribute to the reoccurrence (23). HIF-1 as a sub-type of HIF was a positive relationship with the degree of hypoxia (24). The rapid growth of the tumor may cause tumor inner necrosis, also after feeding arterial embolization. In the group receiving embolization, the expression of HIF-1 is higher than in BEV and control groups.

Moreover, HIF-1 is demonstrated to be elevated in the TACE-treated tumors and the HIF-1 expression was positively relevant to both VEGF level and microvessel density (MVD) in the remaining tumor (25, 26).

Vascular endothelial growth factor (VEGF) is an important angiogenic cytokine that plays a key role in tumor angiogenesis (27). The VEGF signaling pathway also plays an important role in tumor angiogenesis and tumor progression in HCC. Live tumors remaining after liver TACE respond by activating the expression of many hypoxia-responsive genes, inducing persistent expression of vascular endothelial growth factor (VEGF), which leads to the formation of new blood vessels (angiogenesis) and increased microvessel density, ultimately leading to limited therapeutic effect (28, 29). After CB-BEV embolization of the VX**_2_** tumor, the detection of VEGF serum levels showed an increasing trend from post-embolization 1 day to 14 days. While the level of VEGF in the group receiving BEV intravenous application was significantly lower than in the CB-TACE group. However, in the BEV-CB-TACE group, the level of VEGF gradually increased, which may associate with BEV releasing. In addition, the expression of VEGF on tumor tissue is also associated with arterial embolization and BEV application. The results of this study are similar to other experimental results of BEV inhibiting VEGF activity and angiogenesis (14, 16, 30). In this study, the MVD expression is almost positively relevant to the VEGF expression in the liver tumor tissue. While negatively relevant to the HIF-1 expression in tumor received BEV injection and CB-loaded treatment, which contrast to CB-TACE group.

BEV-CB-TACE showed excellent outcomes and safety after embolization. The immunofluorescent staining of TUNEL revealed the number of necrosis tumor cells in the BEV-CB-TACE group was the highest among all 5 groups. Enhanced CT suggested BEV-CB-TACE group has a lower tumor growth rate. Although the levels of ALT, AST, and BUN increased slightly after embolization and returned to the preoperative levels on 7 days, they were all within the normal range. In addition, since the TACE operation of all rabbits was performed by exposing the femoral artery through a skin incision, no wound non-healing was observed, which showed a good application effect.

In addition, this study also has certain limitations. First, the solution to explore BEV release profiles is saline. Compared to blood, protein is lacking, which may affect BEV release profiles *in vivo*. Second, the number of animal models in this study is small. Finally, the liver VX**_2_** tumor model is a squamous cell carcinoma, which exhibits spontaneous central necrosis within the tumor during growth.

## Conclusion

In conclusion, this study confirms that Callispheres beads could efficiency loaded BEV. BEV-CB-TACE has a good safety and effectiveness, and its application could reduce the level of VEGF-A in serum in the treatment of VX**_2_** tumors.

## Data availability statement

The raw data supporting the conclusions of this article will be made available by the authors, without undue reservation.

## Ethics statement

The animal study was reviewed and approved by Zhengzhou University (SYXK202102).

## Author contributions

Conceptualization, KR and XH; investigation, JW, KW, YFL, XG,and ZZ; writing–original draft preparation, KR and YHL; writing review and editing, ZML and JY; and validation, XL and ZL. All authors contributed to the article and approved the submitted version.
